# Waning Humoral Response 6 Month after Double Vaccination with the mRNA-BNT162b2 Vaccine in Hemodialysis Patients

**DOI:** 10.15388/Amed.2023.30.1.3

**Published:** 2023-01-24

**Authors:** Vilma Balčiuvienė, Asta Burčiuvienė, Mathias Haarhaus, Jurgita Uogintaitė, Asta Janavičienė, Lina Santockienė, Jurgita Mitrikevičienė, Loreta Aleknienė, Danutė Keinaitė

**Affiliations:** Diaverum dialysis unit, Diaverum Lithuania, Josvainių 36, LT-57275 Kėdainiai, Lithuania; Diaverum dialysis unit, Diaverum Lithuania, Savanorių 68, LT-44147 Kaunas, Lithuania; Diaverum AB, 21532, Malmö, Sweden; Diaverum Lithuania, Mindaugo 23, LT-3214, Vilnius, Lithuania; Diaverum dialysis unit, Diaverum Lithuania, Žeimių 19, LT-55134 Jonava, Lithuania; Diaverum dialysis unit, Diaverum Lithuania, Beržyno 27, LT-56172 Kaišiadorys, Lithuania; Diaverum dialysis unit, Diaverum Lithuania, S. Neries 20, LT-92228 Klaipėda, Lithuania; Diaverum dialysis unit, Diaverum Lithuania, Savanorių 68, LT-44147 Kaunas, Lithuania; Diaverum Lithuania, Mindaugo 23, LT-3214, Vilnius, Lithuania

**Keywords:** SARS-CoV-2, hemodialysis, antibody titers, vaccination, humoral response

## Abstract

**Introduction:**

Although most hemodialysis patients (HDP) exhibit an initial seroresponse to vaccination against severe acute respiratory syndrome coronavirus 2 (SARS-CoV-2), studies have shown this response to be lower compared to healthy subjects. This fact raised concerns regarding the durability of the immune response and effective protection against severe Coronavirus disease 2019 (COVID-19) in this vulnerable population. The aim of our study was to evaluate the change in antibody levels over time in HDP population.

**Materials and Methods:**

We performed a prospective multicenter study, evaluating antibody response among HDP at 2 and at 6 months after complete two-dose vaccination course with the mRNA-BNT162b2 (Pfizer-BioNTech) vaccine. The study was performed in 14 hemodialysis units of a private dialysis provider in Lithuania. The serum samples of 189 HDP were tested for SARS-CoV-2 IgG against the Spike glycoprotein.

**Results:**

189 HDP participated in the study. Patients were 64.3±15.7 years of age, 116 (61.4%) were males and 73 (38.6%) were females. Among them, 183 (96.8%) were seropositive for anti-S IgG at 2 months after the second immunization dose. Six months after the second dose only 145 (76.7%) of study participants had positive anti-S IgG titers. The median level of anti-S IgG titers after 2 months was 383.1 BAU/mL (166.2–995.6) and after 6 months this level significantly decreased to 51.4 BAU/mL (22.0–104.0) (p<0.001). Seroresponses at both time points inversely correlated with increasing patient’s age. Risk factor for absent response after 2 months included oncologic disease. Systemic autoimmune disease and a history of myocardial infarction increased risk to be seronegative 6 months after the second vaccine dose.

**Conclusions:**

The majority of hemodialysis patients seroresponded after BNT162b2/Pfizer vaccination, but vaccine-induced humoral immunity wanes over time.

## Santrauka.

**Įžanga:**

Nors daugumai hemodializės pacientų (HDP) atsiranda pradinė seroreakcija į skiepus nuo ūminio respiracinio sindromo koronaviruso 2 (SARS-CoV-2), tačiau tyrimai atskleidė, kad šis atsakas yra silpnesnis nei sveikų tiriamųjų. Šis faktas sukėlė susirūpinimą dėl imuninio atsako ir šios pažeidžiamos visuomenės populiacijos efektyvios apsaugos nuo sunkių koronaviruso 2019 (COVID-19) susirgimo atvejų . Mūsų tyrimo tikslas – įvertinti, kaip, bėgant laikui, keičiasi HDP populiacijos antikūnų lygis.

**Medžiagos ir metodai:**

Atlikome perspektyvinį daugiacentrį tyrimą, kuriuo įvertinome antikūnų atsaką HDP populiacijoje praėjus 2 ir 6 mėnesiams po viso dviejų dozių vakcinacijos kurso naudojant mRNA-BNT162b2 (Pfizer-BioNTech) vakciną. Tyrimas buvo atliktas privačios hemodializės paslaugas teikiančios Lietuvos įmonės 14 hemodializės skyrių. 189 HDP serumo mėginiai buvo tiriami nustatyti SARS-CoV-2 IgG smaigalio glikoproteino vertes.

**Rezultatai:**

Tyrime dalyvavo 189 HDP. Pacientų amžius – 64,3±15,7 metų, iš 189 HDP 116 (61,4 %) buvo vyrų, o 73 (38,6 %) moterys. Iš minėto dalyvavusiųjų tyrime skaičiaus 183 (96,8 %) pacientų buvo seropozityvus rezultatas anti-S IgG praėjus dviem mėnesiams po antrosios imunizacijos dozės. Praėjus 6 mėnesiams po antrosios dozės, tik 145 (76,7 %) tyrimo dalyviams buvo nustatyti teigiami anti-S IgG titrai. Anti-S IgG titrų mediana praėjus dviem mėnesiams buvo 383,1 BAU/mL (166,2–995,6), o po 6 mėnesių šis lygis reikšmingai sumažėjo iki 51,4 BAU/mL (22,0–104,0) (p < 0,001). Abiem tyrimo momentais seroreakcijos vertės atskleidė atvirkštinę koreliaciją su didėjančiu paciento amžiumi. Rizikos veiksnys nesant atsako po dviejų mėnesių taip pat apėmė ir onkologinės ligos atvejį. Sisteminė autoimuninė liga ir sveikatos istorijoje esantis miokardo infarktas padidino seronegatyvaus atsako riziką praėjus 6 mėnesiams po antrosios vakcinos dozės.

**Išvados:**

Daugumai hemodializės pacientų buvo seroreakcija po BNT162b2/Pfizer vakcinacijos, tačiau vakcinos sukeltas humoralinis imunitetas laikui bėgant mažėja.

## Introduction

Maintenance hemodialysis patients (HDP) have been identified as having higher morbidity and mortality after severe acute respiratory syndrome coronavirus 2 (SARS-CoV-2) infection, owing to their defective immunity and comorbidities. The reported prevalence of COVID-19 among HDP ranged from 5.3% to 36.2%, hospitalization rates have been 3-to-4 fold higher compared with the general population and a mortality is exceeding 20% [1-8]. To reduce the poor prognosis in that vulnerable population, HDP were prioritized for vaccination in many countries [9-11]. But no patients with end stage renal disease (ESRD) have been included in randomized and controlled trials of COVID-19 vaccines. Given the fact that HDP respond less well to vaccines than the general population due to the impaired function of the immune system [12-15], it is uncertain, whether vaccination against COVID-19 will result in sufficient immune response as it was demonstrated in clinical studies with healthy individuals [16,17].

We aimed to investigate the robustness and longevity of immune response in ESRD patients. For that purpose we conducted a prospective multicenter study to assess vaccine-induced antibody response in this population and evaluate the humoral immunity status over time.

## Materials and methods

### Patients

This was a prospective multicenter study, performed in 14 hemodialysis units of a private hemodialysis provider in Lithuania. This dialysis provider is one of the largest providers of hemodialysis services in Lithuania and perform HD procedures for 350 patients. In total, there are about 1,200 hemodialysis patients in Lithuania. Thus, patients of this private HD provider account for about one quarter of all hemodialysis patients in Lithuania.

Patients were considered eligible if they were on chronic dialysis for at least 1 month and had completed a two-dose vaccination course with the mRNA BNT162b2 (Pfizer-BioNTech) vaccine, with a 3-week interval between the first and the second dose from January 5 to March 09, 2021. The study was approved by the Lithuanian Bioethics Committee and conducted according to the guidelines of the Declaration of Helsinki.

For each patient, the following demographic, clinical and laboratory parameters were collected as recorded in the medical files: age, sex, body mass index (BMI), weekly hemodialysis (HD) duration, time on dialysis, HD access, primary kidney disease, comorbidities, medications, allergies, addictions, hospitalizations in a 12-month period, response to hepatitis B vaccination, lymphocyte and platelet count, hemoglobin, ferritin, parathyroid hormone, C-reactive protein (CRP), urea, serum creatinine, albumin, protein, calcium, phosphate and cholesterol levels, Kt/v_urea_ (clearance of urea multiplied by dialysis duration and normalized for urea distribution volume). The listed laboratory parameters were performed on a scheduled basis (once a month) and evaluated in the same month as the vaccination. Each patient was interviewed about side effects and adverse reactions related to vaccination – pain at injection site, fever, myalgia, nausea, vomiting, headache and general weakness. These data were documented in the medical files. Hospitalizations were of any other reason except COVID-19 infection.

### Anti-SARS-CoV-2 anti-S IgG antibody analysis

Blood samples were collected before the start of HD session. Antibody response among HDP was evaluated twice, at 2 and 6 months after receiving the second dose of BNT162b2 (Pfizer-BioNTech). Serum was tested for SARS-CoV-2 IgG against the Spike glycoprotein (anti-S IgG) using an immunoassay analyzers Siemens ADVIA Centaur XP/Atellica/sCOVG assay traceability to WHO 20/136. According to the manufacturer’s recommendations Binding Antibody Units per milliliter (BAU/mL) ratio of <21.8 was considered as negative.

### Statistical analysis

Statistical analysis of demographic and laboratory variables was performed using descriptive statistics. The difference between two independent groups was tested using Mann–Whitney–Wilcoxon test. The relation between the qualitative values has been evaluated following the Pearson chi-square criteria. The statistical significance for group comparisons was set at p<0.05. Univariate and multivariate logistic regression analyses were performed to explore factors associated with negative antibody response. The data analysis was performed with the software “Statistical Package for Social Sciences” (SPSS version 22.0).

## Results

### Characteristics of study population

Initially, 314 dialysis patients were recruited for the study. 111 patients who had been infected with COVID-19 before or after vaccination were excluded. Six patients were excluded from the study because inpatient treatment was carried out 2 months after vaccination and serum samples for anti-S IgG were not available. Four patients were excluded because they passed away and 4 study participants were excluded due to undergoing transplantation. Finally, we enrolled 189 patients. In order to substantiate representativeness of the data, the Paniotto sample calculation formula was applied, which enabled to obtain 95 percent of reliability degree (the desired accuracy is 5 percent), i.e. n = 189 was enough for reliability.

The group of patients participating in the study consisted of 116 (61.4%) males and 73 (38.6%) females, the median (MD (Q1–Q3)) age 66.0 (55.5–76.5) years. Median time on hemodialysis was 4 years (2–7.4). Two months after the second immunization dose, 183 (96.8%) of the 189 hemodialysis patients were seropositive for anti-S IgG. Six months after the second dose the number of seropositive patients significantly decreased – seropositive for anti-S IgG were only 145 (76.7%) of study participants (p<0.001). The baseline characteristics of all HDP, responders and nonresponders after 2 and 6 months are detailed in Table 1.

**Table 1.** Clinical characteristics and immune response of 189 hemodialysis patients (HDP) after two doses of the mRNA-based SARS-CoV-2 vaccine BNT162b2

		**After 2 months**			**After 6 months**		
**Variable name**	**All (n=189)**	**Responders (n=183)**	**Non-responders (n=6)**	**p value**	**Responders (n= 145)**	**Non- responders (n=44)**	**p value**
**Age (years), median(Q1-Q3)**	66.0(55.5-76.5)	66.0(55.0-77.0)	59.5(57.8-72.3)	0.063	65.0(53,0-75.0)	70.0(60.3-80.8)	0.014
**Male gender, n(%)**	116(61.4)	114 (62.3)	2 (33.3)	0.152	90 (62.1)	26(59.1)	0.722
**BMI(kg/m^2^), median(Q1-Q3)**	26.0(23.4-30.4)	26.0(23.3-30.4)	29.0(23.0-39)	0.250	26.1(23.2-30.4)	25.6(23.6-30.0)	0.893
**Dialysis vintage, (years), median(Q1-Q3)**	4(2.0-7.4)	4.0(2.0-7.1)	4.0(2.8-11.0)	0.742	4.0(2.0-7.0)	5.0(3.0-7.5)	0.202
**A-v fistula as dialysis access, n(%)**	166(87.8)	161(88)	5(83.3)	0.732	127 (87.6)	39 (88.6)	0.852
**Adicction n(%)**	28(14.8)	27(14.8)	1(16.7)	0.897	19(13.1)	9(20.5)	0.229
**Primary kidney disease, n(%)**							
Diabetic nephropathy	24(12.7)	23(12.6)	1(16.7)	0.987	17(11.7)	7(15.9)	0.749
Hypertensive kidney disease	50(26.5)	48(26.2)	2(33.3)	0.987	35(24.1)	15(34.1)	0.749
Glomerulonephritis	33(17.5)	32(17.5)	1(16.7)	0.987	25(17.2)	8(18.2)	0.749
ADPKD	26(13.8)	25(13.7)	1(16.7)	0.987	22(15.2)	4(9.1)	0.749
Pyelonephritis	24(12.7)	23(12.6)	1(16.7)	0.987	19(13.1)	5(11.4)	0.749
Tubulointerstitial disease	9(4.8)	9(4.9)	0	0.987	7(4.8)	2(4.5)	0.749
Other	18(9.5)	18(9.8)	0	0.987	16(11.0)	2(4.5)	0.749
**Comorbidity, n(%)**							
Diabetes mellitus	37(20.5)	36(19.7)	1(16.7)	0.729	28(19.3)	9(20.5)	0.556
Hypertension	162(85.7)	158(86.3)	4(66.7)	0.205	124(85.5)	38(86.4)	0.888
Ischemic heart disease	90(47.6)	87(47.5)	3(50)	1.000	65(44.8)	25(56.8)	0.163
History of malignancy	29(15.3)	25(13.7)	4(66.7)	0.006	20(13.8)	9(20.5)	0.283
Systemic autoimmune disease	12(6.3)	11(6.0)	1(16.7)	0.329	6(4.1)	6(13.6)	0.024
Cerebrovascular disorder	32(16.9)	32(17.5)	0	0.261	29(20)	3(6.8)	0.041
Peripheral vascular disease	37(19.6)	37(20.2)	0	0.219	30(20.7)	7(15.9)	0.484
**Medication, n(%)**							
Antihipertensive	153 (81)	150(81.9)	3(50)	0.084	114(78.6)	39(88.6)	0.138
Oral hypoglycemic drugs and/or Insulin	25(13.2)	24(13.1)	1(16.7)	0.578	17(11.7)	8(18.2)	0.268
Anticoagulant	47(24.9)	44(24.0)	3(50)	0.164	37(25.5)	10(22.7)	0.708
Anti-cancer	5(2.6)	5(2.7)	0	1.0	5(3.4)	0	0.592
Glucocorticoids	5(2.6)	5(2.7)	0	1.0	3(2.1)	2(4.5)	0.331
Nonsteroidal anti-inflammatory drugs	30(15.9)	28(15.3)	2(33.3)	0.243	20(13.8)	10(22.2)	0.155
Proton pump inhibitors	49(25.9)	46(25.1)	3(50)	0.181	34(23.4)	15(34.1)	0.158
Statins	36(19)	34(18.6)	2(33.3)	0.321	27(18.6)	9(20.5)	0.786
Antiviral	2(1.1)	2(1.1)	0	1.0	2(1.4)	0	1.0
Others (erythropoetin, intravenous iron)	77(40.7)	74(40.4)	3(50)	0.689	66(45.5)	11(25)	0.015
**Hospitalization (in 12 months), n(%)**	73(38.6)	69(37.7)	4(66.6)	0.208	54(37.2)	19(43.2)	0.478
**Hepatitis B nonresponder, n(%)**	37(19.6)	34(18.6)	3(50)	0.274	29(20)	8(18.2)	0.812

BMI – body mass index, ADPKD – autosomal dominant polycystic kidney disease.

The laboratory findings of our patients are detailed in Table 2.

**Table 2.** Laboratory findings in 189 hemodialysis patients (HDP).

**Laboratory tests**	**Median (Q1-Q3)**
Lymphocytes count (x10*9/l)	1.42 (1.1-1.86)
Hemoglobin (g/l)	117 (110-123)
Platelets count (x10*9/l)	224 (176-277.3)
Ferritin level (mcg/l)	367.3 (271.3-474.9)
CRP (mg/l)	4.85 (1.26-10.28)
BUN (mmol/l)	22.1 (18.2-25.4)
Serum creatinine (mcmol/l)	707 (552-832)
Kt/v	1.61 (1.45-1.76)
Calcium (mmol/l)	2.36 (2.27-2.44)
Phosphorus (mmol/l)	1.64 (1.35-2.0)
PTH (pmol/l)	30.2 (15.0-53.4)
Total protein (g/l)	68.0 (64.0-71.0)
Albumin (g/l)	41.2 (39.35-43.6)
Cholesterol (mmol/l)	4.73 (4.05-5.41)

CRP – C-reactive protein, PTH – parathyroid hormone, BUN – blood urea nitrogen, Kt/v – clearance of urea multiplied by dialysis duration and normalized for urea distribution volume

### Predictors of SARS-CoV-2 anti-S IgG levels

**Figure 1.**
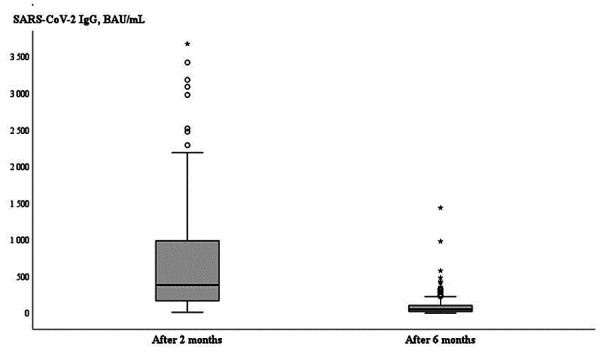
Comparison anti-S IgG (BAU/ml) at 2 and 6 months after second dose of BNT162b2 vaccine in HDP patients (n=189).

The median level of anti-S IgG titer after 2 months was 383.1 BAU/mL (166.2–995.6) and after 6 months this level significantly decreased to 51.4 BAU/mL (22.0–104.0) (p<0.001) (Figure 1).

p<0.001 by Wilcoxon Test

**Figure 2.**
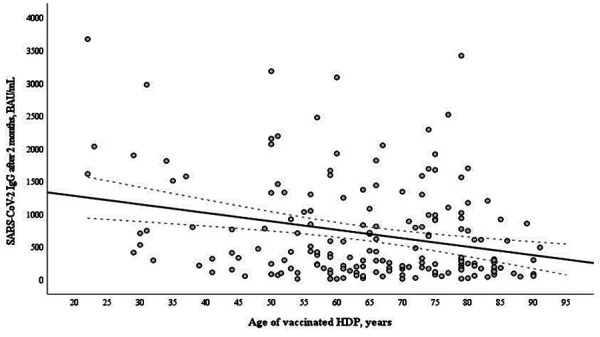
Correlation of age (years) and anti-S IgG (BAU/mL) in 189 hemodialysis patients (HDP) at 2 months after two doses of the mRNA-based SARS-CoV-2 vaccine BNT162b2 (r=-0,205, p=0,006).

**Figure 3.**
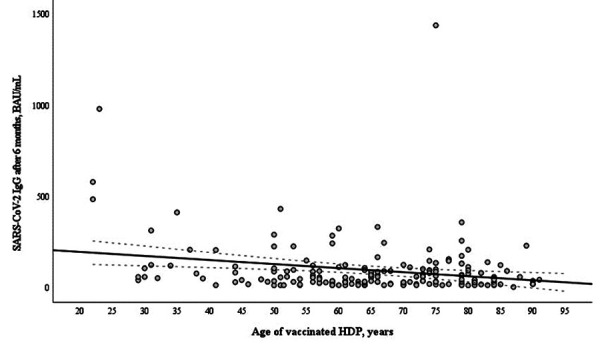
Correlation of age (years) and anti-S IgG (BAU/mL) in 189 hemodialysis patients (HDP) at 6 months after two doses of the mRNA-based SARS-CoV-2 vaccine BNT162b2 (r=-0.148, p=0.049).

Six months after vaccination median age of seronegative patients was significantly higher than that of seropositive – 70.0 years (60.3–80.8) vs 65.0 years (53.0–75) (p=0.014). Meanwhile, there was no significant difference in age between responders and nonresponders 2 months after vaccination The antibody responses in hemodialysis patients negatively correlated with age at 2 months (Figure 2) and at 6 months (Figure 3) after the second vaccine dose.

For further analysis of the age effect, HDP were subdivided in to age-related groups: <55, 56–65, 66–75 and ≥75 years. Six months after the second vaccination dose the prevalence of negative antibody response was significantly higher in patients aged of >75 years compared to the younger patients aged of ≤55 years – 17 (35.4%) vs 7 (14.9%) (p<0.05). According to logistic regression analysis, we have established that patients of age >75 years compared to those of <55 years increased odds ratio (OR) to be seronegative for anti-S IgG 6 months after second vaccine dose by 3.134 times [95% CI 1.156–8.496, p=0.025]. Meanwhile, 2 months after vaccination there was no difference between the patients‘ age group and the seroresponse.

We did not find any significant relation between anti-S IgG titres and sex, BMI, HD access, time on dialysis, weekly HD duration, primary kidney disease, allergies, addictions, hospitalizations in a 12-month period, and antibody production at any time point. Use of other medications (erythropoetin, intravenous iron) at 6 months after the second dose was significantly more often in responders group if compared to nonresponders (45.5% vs 25%, p=0.015). At 2 months after vaccination this difference was not observed.

An oncologic disease increased the risk to be seronegative for anti-S IgG at 2 months after the second vaccine dose (OR 12.64 [95% CI 2.199–72.665, p=0.006]). A history of myocardial infarction (OR 2.352 [95% CI 1.013–5.46, p=0.042]) and systemic autoimmune disease (OR 3.658 [95% CI 1.116–11.989, p=0.024]) increased the risk to be seronegative at 6 months after the second vaccine dose, while a history of cerebrovascular disease was associated with a decreased risk at 6 months (OR 0.293 [95% CI 0.085–1.012, p=0.041]).

**Figure 4.**
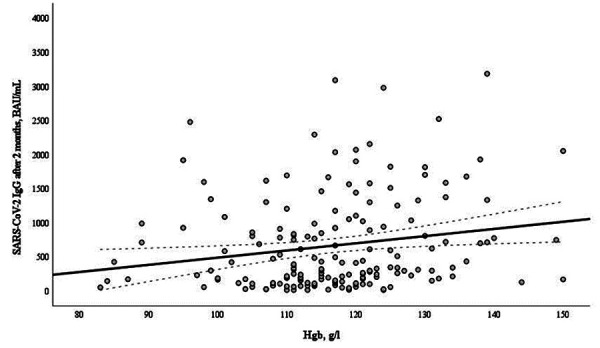
Correlation of hemoglobin level (g/l) and anti-S IgG (BAU/mL) in 189 hemodialysis patients (HDP) after two doses of the mRNA-based SARS-CoV-2 vaccine BNT162b2 (r=0.206, p=0.007).

At 2 months after second vaccine dose, there was a significant positive correlation between hemoglobin level and serological response (Figure 4). At 6 months after vaccination there was no correlation between hemoglobin and anti-S IgG levels.

According to logistic regression analysis we have established that at 6 months after the second dose, OR to be seronegative for anti-S IgG for patients whose hemoglobin <120g/l was 2.921 [95% CI 1.334–6.396, p=0.006]. There was no significant association between other laboratory parameters (lymphocyte and platelet count, ferritin level, CRP, urea, serum creatinine, Kt/v, calcium, phosphate, parathyroid hormone, total protein, albumin, cholesterol levels) and anti-S IgG levels.

Multivariate regression analysis revealed that higher age, hemoglobin below 120 g/l, and use of other medications were independent predictors of a negative antibody response at 6 months after the second vaccine dose (Table 3).

**Table 3.** Multivariate regression analysis: Independent factors of a negative antibody response.

**Variables**	**Odds ratio**	**95% confidence interval**	**p value**
Higher age	2.267	1.091 – 4.710	0.028
Hemoglobin <120 g/l	2.778	1.244 – 6.203	0.013
Other medications	2.616	1.183 – 5.785	0.018

### Tolerability of BNT162b2 in hemodialysis patients

After the first dose of BNT162b2 injection, only mild localized pain at the injection-site was reported by some HDP. Fever and myalgia occured more frequently after the second vaccine dose. Frequencies of reactions to the vaccine are summarized in Table 4. There was no difference of adverse reactions between responders and nonresponders. Overall, the vaccination was well tolerated.

**Table 4.** Frequency of adverse reactions in hemodialysis patients (HDP) after vaccination with mRNA-based SARS-CoV-2 vaccine BNT162b2.

**Adverse reactions**	**After first dose**	**After second dose**	**P value**
Pain at injection site	11.6	6.3	0.036
Fever	1.1	12.7	<0.001
Myalgia	0	7.9	<0.001
Nausea, vomiting	1.1	0.5	0.281
Headache	1.1	2.1	0.205
General weaknes	1.1	4.2	0.027

## Discussion

This study reports the humoral response at 2 and 6 months after vaccination with mRNA-based vaccine BNT162b2 against SARS-CoV-2 in maintenance HDP. The majority of patients in this study responded well to BNT162b2 vaccine with a seroconversion of 96.8% at 2 months after vaccination, but over time the humoral response decreased.

In the HDP population, presented in the current study, the seroconversion rates at 2 months after the second vaccine dose was similar to previous reports [18-33]. Initial studies demonstrated a substantial humoral response in the dialysis population, with seroconversion rates as high as 96%, though levels of antibody titers were significantly lower and more delayed compared to healthy controls [18-26, 34]. Direct comparison of antibody titers at month 2 to those reported in previous studies is difficult, because different antibody assays were used with different definition of the immune response, titers assessed at different time points (mostly within a month after vaccination).

Lower antibody titers observed in the early postvaccination period indicated a worrisome prevalence of attenuated response among dialysis patients after immunization. In addition, immunity is known to decrease after natural COVID-19 infection in patients with hemodialysis treatment [35-37]. These facts raised concerns that the immune response to vaccination will continue to decline or disappear over time.

In this current study we showed highly reduced SARS-CoV-2 antibody titres at 6 months after a second vaccine dose in HDP. Only 76.7% of study participants were positive for anti-S IgG at 6 months after vaccination. Some other published reports showed waning humoral immunity against SARS-CoV-2 after standard scheme of vaccination with BNT162b2 in HDP as well [38-42].

We believe that our study provides strong evidence that the majority of patients undergoing maintenance hemodialysis require booster vaccination. More important, protection offered by first generation vaccines is reduced for SARS-CoV-2 variants of concern, which now represent the majority of global infections [43]. It’s possible, that significantly higher antibody activity is required to prevent breakthrough infections.

We identified risk factors for nonresponders, low and high responders to vaccination. Older age was associated with lower antibody levels at 2 and 6 months after full vaccination with BNT162b2 and loss of response at 6 months after vaccination. Negative correlation between older age and antibody response was shown in many other studies, which examined the relationship between age and the strength of immunity. It is likely that older people require booster vaccination or adapted vaccination schedule, to increase the effectiveness of an mRNA vaccine. Interestingly, we found that oncologic disease was associated with non-response at 2 months after second BNT162b2 vaccine dose, but number of nonresponders didn’t increase significantly at 6 months after vaccination. It’s possible that oncologic disease leads to a failure of the immune response to vaccination, but it doesn’t influense the rapid loss of the immune response over time. Past history of myocardial infarction and systemic autoimmune disease was associated with antibody loss at 6 months after full vaccination with BNT162b2. Therefore attention should be paid to this group of patients due to the possible rapid loss of immune response.

There was a significant positive correlation between hemoglobin level and serological response at 2 months after vaccination, but there was no significant correlation between these factors and immune response at 6 months after vaccination.

We didn’t find any significant correlations between other investigated factors, such as gender, BMI, dialysis vintage, dialysis access, addictions, primary kidney disease, hospitalizations, hepatitis B nonresponders, Kt/v, other laboratory findings, and humoral response to vaccination with BNT162b2. We want to single out the fact that vaccination with BNT162b2 elicited immune response to those patients who didn‘t respond to the hepatitis B vaccine Engerix-B, which is given in double doses as a four-series vaccine. And such risk factors, like diabetes, obesity, dialysis vintage, malnutrition, or inflammation, which impair protective immunity after hepatitis B vaccinations, didn‘t influence response to vaccination with BNT162b2 [44-47]. It seems that the type of antigenic presentation or stimulation, conducted by mRNA vaccines, may overcome uremia-induced immune alterations, but is not strong enough to generate the same immune response as in healthy individuals.

In many studies immunosuppression or chemotherapy are an unfavorable factors for antibody formation, but we couldn’t evaluate this relation as we didn’t have such treated patients.

Vaccination with BNT162b2 was well tolerated and safe, as it was reported in phase 3 clinical trials for BNT162b2 [16] and in most studies with ESRD patients. Only mild localized pain at the injection site, myalgia and fever were frequently reported by the maintenance dialysis patients. Hemodialysis patients exhibited lower local and systemic adverse events compared to healthy population. Pain at the injection site was reported by less than 12% of hemodialysis patients, whereas that was reported in 50% to 60% of healthy controls in some studies. Systemic adverse effects, such as fever, general weakness, headache, myalgia, nausea were reported in less than 13% of patients with MHD, whereas that was reported in 20% to 30% of healthy controls [20]. Fever and myalgia was much more expressed after the second dose of BNT162b2. No serious side effects were observed in our study.

There are several strengths of the present study. First, the study was performed in a controlled, prospective manner with the same protocol including time points of sampling and analysis in dialysis patients. Second, samples from all patients in 14 hemodialysis centers were analyzed in the same laboratory using an identical protocol for quantification of antibody levels.

This study has several limitations. First, not all patients were tested for antibodies against SARS-CoV-2 before enrolment to study. Therefore we cannot completely exclude the possibility that the seroconversion may reflect previous asymptomatic infection versus vaccination in some HDP. In 2021 January and February vaccination was rushed to protect HDP from severe COVID-19. On the contrary, the procedures for obtaining a research permit by the Lithuanian Bioethics Committee took about 2 months, so some patients were not tested for antibodies prior to enrollment in the study. Until the start of vaccination patients were tested for COVID-19 routinely, so we think that the likelihood of asymptomatic COVID-19 cases before vaccination start is very low. Second, we did not compare results of HDP with a control group of healthy people. Third, we didn‘t analyze the cellular response to vaccination. The sole analysis of humoral response may underestimate or overestimate the immunogenicity of the vaccine, as the cellular part of immune system plays a great role here.

Six months after 2 doses of BNT162b2 hemodialysis patients were given the 3rd dose of the same vaccine according to the national vaccination policy in Lithuania. Whether booster dose will induct long lasting humoral immunity and adequate protection from severe COVID-19 in this vulnerable population requires further research. We continue to monitor the immune response to the 3rd dose of BNT162b2 in HDP in 14 hemodialysis units of the private healthcare center Diaverum in Lithuania. We hope that the data will help in defining the future vaccination strategy for our patients.

## Conclusions

Seropositivity rates following BNT162b2 vaccination in hemodialysis patients were high. Overall, 183 hemodialysis patients (96.8%) were seropositive for SARS-CoV-2 anti-S IgG at 2 months following the second dose of BNT162b2 vaccination. Six months after second vaccine dose HD patients presented with highly reduced SARS-CoV-2 antibody titres. Older patients were less likely to present an antibody response at 2 and 6 months after vaccination with BNT162b2. Oncologic disease was associated with lack of response 2 months after second BNT162b2 vaccine dose. Past history of myocardial infarction and systemic autoimmune disease were associated with antibody absence at 6 months after the second vaccine dose. At 2 months after second vaccine dose there was a significant positive correlation between hemoglobin level and serological response. Use of erythropoetin and intravenous iron correlated with better humoral response at 6 months after the second BNT162b2 vaccine dose.
